# Preoperative nutritional status as a predictor of postoperative overall survival in abdominal tumor surgery: a systematic review and meta-analysis

**DOI:** 10.3389/fsurg.2025.1645392

**Published:** 2025-08-18

**Authors:** Zhaoyin Su, Yifeng Lin, Molan Li, Yanyan Yang, Xiaohan Chen, Yifu Zhu, Yifan Mo, Zhirui Huang, Yatao Liu, Nerlich Michael

**Affiliations:** ^1^ The First School of Clinical Medicine, Lanzhou University, Lanzhou, China; ^2^ School of Philosophy and Sociology, Lanzhou University, Lanzhou, China; ^3^ The Second School of Clinical Medicine, Lanzhou University, Lanzhou, China; ^4^ School of Mathematics and Computer Science, Shantou University, Shantou, China; ^5^ Faculty of Engineering, The Chinese University of Hong Kong, Hong Kong SAR, China; ^6^ Department of Anesthesia, First Hospital of Lanzhou University, Lanzhou, China; ^7^ Department of Trauma Surgery, University Hospital Regensburg, Regensburg, Germany

**Keywords:** preoperative nutritional status, abdominal tumor surgery, postoperative overall survival, enhanced recovery after surgery, surgery, meta-analyses

## Abstract

**Background:**

Abdominal tumors, including those in the stomach, colon, pancreas, and gallbladder, significantly impact global morbidity and mortality. Surgical resection is the primary treatment, but postoperative outcomes and long-term survival are often affected by factors such as preoperative nutritional status. Malnutrition is common in these patients, making its management crucial for improving outcomes. This systematic review and meta-analysis aim to consolidate evidence on the role of preoperative nutritional status in postoperative survival for patients undergoing abdominal tumor surgery, offering insight into its prognostic value.

**Methods:**

A systematic literature search was conducted using electronic databases to report the impact of the preoperative nutritional status on OS (overall survival) of patients with abdominal tumor surgery as of January 1st, 2025. The hazard ratio (HR) with a 95% confidence interval (CI) was used to evaluate the impact of the preoperative nutritional status on OS.

**Results:**

A total of 32 studies involving 10352 patients were included in the meta-analysis. The results (pooled HR: 1.61, 95% CI: 1.49–1.73, *I*² = 43.0%, *p* < 0.001) indicated that preoperative malnutrition is significantly associated with poorer OS. Subgroup and meta-regression analyses based on methods of nutritional status assessment, country, sample size, study design, follow-up duration, analytical model, and tumor type all showed a consistent association between preoperative malnutrition and worse OS. The robustness of these pooled results was further verified through sensitivity analysis. Additionally, the heterogeneity of pooled HR of OS was attributed to differences in study designs, as indicated by meta-regression analysis (*p* = 0.005). Funnel plots did not show significant publication bias.

**Conclusion:**

Based on existing evidence, the preoperative nutritional status is a valuable predictor of postoperative OS in patients with abdominal tumor surgery.

**Systematic Review Registration:**

PROSPERO CRD420251008979.

## 1 Introduction

Abdominal tumors, primarily consisting of gastrointestinal malignancies such as gastric cancer, colorectal cancer, pancreatic cancer, and liver cancer, are often associated with varying degrees of malnutrition and muscle wasting (1–3). These conditions are typically characterized by high mortality rates, primarily due to the subtle onset of symptoms, with many patients being diagnosed at advanced stages (4–7). Given that the majority of these cancers are diagnosed at later stages, treatment options are limited, and surgical resection remains the primary therapeutic approach (8, 9). Therefore, improving postoperative outcomes for these patients is crucial to enhancing their overall health and well-being.

Cancer patients often present with complex conditions and multiple comorbidities, with numerous factors influencing their prognosis (10). Identifying more controllable, simple factors that can improve postoperative outcomes is essential. Since the 1990s, numerous studies have highlighted the widespread issue of poor nutritional status among cancer patients, which has been associated with unfavorable postoperative outcomes (11–13). The growth of abdominal tumors and symptoms such as anorexia can impair gastrointestinal function, leading to malnutrition (14). Surgical interventions further increase metabolic demands, exacerbating pre-existing nutritional deficiencies (15). Research indicates that malnutrition not only compromises immune function but may also result in slower postoperative recovery, increased complication rates, and prolonged hospitalization (16, 17). Therefore, early assessment and intervention of the preoperative nutritional status may have a positive impact on the postoperative prognosis of patients undergoing abdominal cancer surgery.

Previous studies have demonstrated that preoperative malnutrition is associated with adverse postoperative outcomes in cancer patients (18). However, many of these studies rely on subjective questionnaires to assess nutritional status (19–21), which may introduce biases such as communication difficulties, recall errors, social desirability biases, and comprehension issues, potentially affecting the accuracy of the results. This underscores the need for more objective tools to assess the risk of malnutrition.

In response to this need, several objective nutritional assessment tools have been proposed, offering a more accurate and reliable means of evaluating nutritional status. These tools include the Prognostic Nutritional Index (PNI) (22), the Controlling Nutritional Status (CONUT) score (23), and the Geriatric Nutritional Risk Index (GNRI) (24). PNI, which includes serum albumin levels and lymphocyte count, has been widely used to assess immune-nutritional status and predict postoperative outcomes in gastrointestinal cancers (22). The CONUT score, a system for evaluating nutritional status, incorporates serum albumin levels, total lymphocyte count, and total cholesterol levels, and has become an important prognostic tool for patients undergoing abdominal tumor resections, including pancreatic cancer, liver cancer, and other abdominal cancers (23). The GNRI, calculated using serum albumin levels and the ratio of ideal to actual body weight, has also been identified as a key predictor of overall survival in patients undergoing abdominal tumor resections (25).

In the context of modern oncologic surgery, the emergence of the Enhanced Recovery After Surgery (ERAS) program further supports the integration of nutritional assessment into preoperative evaluation. These multidisciplinary pathways, which emphasize early mobilization, pain control, and nutritional support, have been shown to improve postoperative outcomes and shorten hospital stays (26). Despite the substantial evidence linking poor nutritional status with increased postoperative complications and reduced survival rates, the current literature remains fragmented, with variations in assessment methods and outcome measures.

To address the gap in this field, our systematic review and meta-analysis aim to evaluate the prognostic role of preoperative nutritional status on overall survival (OS) in patients undergoing abdominal tumor surgery, based on objective nutritional assessment tools. This study seeks to support clinical risk stratification and intervention strategies, provide a theoretical foundation for future research, and ultimately optimize the management of patients undergoing abdominal tumor surgery, thereby improving their long-term outcomes.

## 2 Materials and methods

This systematic review and meta-analysis was conducted following the Preferred Reporting Items for Systematic Reviews and Meta-Analyses (PRISMA) statement (27). The study is registered with PROSPERO under registration number CRD420251008979.

### 2.1 Search strategies

PubMed, Embase, Web of Science, and Cochrane Library databases were searched for eligible articles up to January 1st, 2025. The search was conducted using medical subject headings (MeSH) in combination with free text words. The search strategy in PubMed database was the following: (nutritional status[MeSH Terms] OR malnutrition[MeSH Terms) AND (preoperative malnutrition[Title/Abstract] OR preoperative dystrophy[Title/Abstract] OR preoperative GNRI[Title/Abstract] OR preoperative PNI[Title/Abstract] OR preoperative CONUT[Title/Abstract) AND prognosis[Title/Abstract]. The search strategies used in all databases are available in Supplementary File S1. In the study selection phase, blinding was implemented to reduce bias and ensure the objectivity and accuracy of the study selection process.

### 2.2 Inclusion criteria

The selection criteria for this research adhere to the PICOS framework (Population, Intervention/Exposure, Comparator, Outcomes, Study Designs). Studies meeting these criteria will be included, with no restrictions regarding language or publication date.

#### 2.2.1 Population

Adult patients (18 years or older) who have undergone abdominal cancer surgery, with no exclusions based on nationality, race, ethnic background, gender, or professional status.

#### 2.2.2 Exposure

Preoperative nutritional status or preoperative nutritional assessment, specifically based on the PNI, CONUT, or GNRI, to identify patients diagnosed with malnutrition.

#### 2.2.3 Comparator

Adults with normal preoperative nutritional status, when applicable.

#### 2.2.4 Primary outcome

Postoperative overall survival.

#### 2.2.5 Study designs

Randomized controlled trials, cohort studies, case-control studies, and observational studies.

### 2.3 Exclusion criteria

This research will exclude studies involving non-adult patients (under 18 years of age) and those with incomplete data, including insufficient or missing preoperative nutritional assessment information, or those not reporting the specified outcomes, such as postoperative overall survival. Studies focusing on patients with non-abdominal cancers, including those undergoing surgery for thoracic, brain, or other non-abdominal malignancies, will also be excluded. Additionally, studies that concentrate on postoperative nutritional interventions or outcomes, rather than preoperative assessments, will not be considered. Research articles lacking comprehensive clinical data, even after repeated attempts to contact the authors, will be excluded. Correspondence, conference abstracts, editorial pieces, case studies, review articles, and any studies that fail to provide sufficient clinical data will also be excluded. Finally, full-text scholarly works that are inaccessible despite thorough search efforts will not be included.

### 2.4 Data extraction

Two investigators (ZYS and XHC) independently extracted the necessary data from the included studies, and any disagreements were resolved through discussion until a consensus was reached. The following data were extracted from each study: first author, publication year, country, study type, study design, sample size, male/female distribution, tumor type, surgical procedure, duration of follow-up, postoperative chemotherapy, overall survival (OS) with hazard ratio (HR) and its 95% confidence interval (CI), type of analysis and the cutoff values for nutritional status scores. In cases where both univariate and multivariate analyses were performed, multivariate analysis was preferred for obtaining HRs for OS, due to adjustments for confounding factors. If HR with 95% CI was not provided in the original studies, the data were extracted from the survival curve using Engauge Digitizer software (28). During the entire data extraction phase, repeated extraction procedures were implemented to ensure the objectivity and accuracy of the data.

### 2.5 Quality assessment

The Newcastle-Ottawa Quality Assessment Scale (NOS) was used to assess the methodological quality of the included studies (29). The NOS evaluates studies across three key domains: selection (with a maximum score of 4 points), comparability (with a maximum score of 2 points), and outcomes (with a maximum score of 3 points). Studies that achieved a score of six or higher were considered to be of high quality (30). This assessment was carried out independently by two investigators (YFL and XHC) to ensure the reliability and objectivity of the evaluation process. When there was a difference, the disagreement was resolved through discussion with a third investigator (YFZ) until consensus was reached. The detailed results of the quality assessment can be found in Supplementary Table S1.

### 2.6 Statistical analysis

Statistical analyses and graphical representations were conducted using R 4.3.3 and STATA 16.0. Pooled HRs with 95% CIs were calculated to evaluate the association between preoperative nutritional status and postoperative OS in patients undergoing abdominal cancer surgery. Heterogeneity among the studies was assessed using the chi-square test and I² statistic. If no significant heterogeneity was detected (*P* ≥ 0.10 or *I*² ≤ 50%), a fixed-effect model was applied for the meta-analysis. In the presence of significant heterogeneity (*I*² > 50% or *P* < 0.10), a random-effects model was employed.

To explore and account for heterogeneity across studies, subgroup analyses, meta-regression, and sensitivity analyses were performed. The subgroup factors included:

1. Preoperative nutritional status, categorized based on the PNI, CONUT, and GNRI scores.
2. Study country (China vs. Japan).
3. Sample size (<200 vs. ≥200).
4. Study design (Multicenter vs. Single-center).
5. Follow-up duration, comparing studies with clearly defined median or average follow-up times to those without.
6. Type of analysis (Univariate vs. Multivariate).
7. Tumor type, categorized as Cholangiocarcinoma, Gallbladder cancer, Renal cancer, Colorectal cancer, Liver cancer, Gastric cancer, or Pancreatic cancer.

If the original studies included in the research have excessive missing data for certain clinical variables (such as the duration of follow-up and postoperative chemotherapy), subgroup analysis will be conducted based on whether the study lacks data for those variables, rather than grouping based on the variable values. Furthermore, publication bias was visually assessed using a funnel plot and quantitatively examined using Begg's and Egger's tests. All statistical tests were two-sided, and *P*-values less than 0.05 were considered statistically significant.

## 3 Results

### 3.1 Study selection

**Figure 1:**
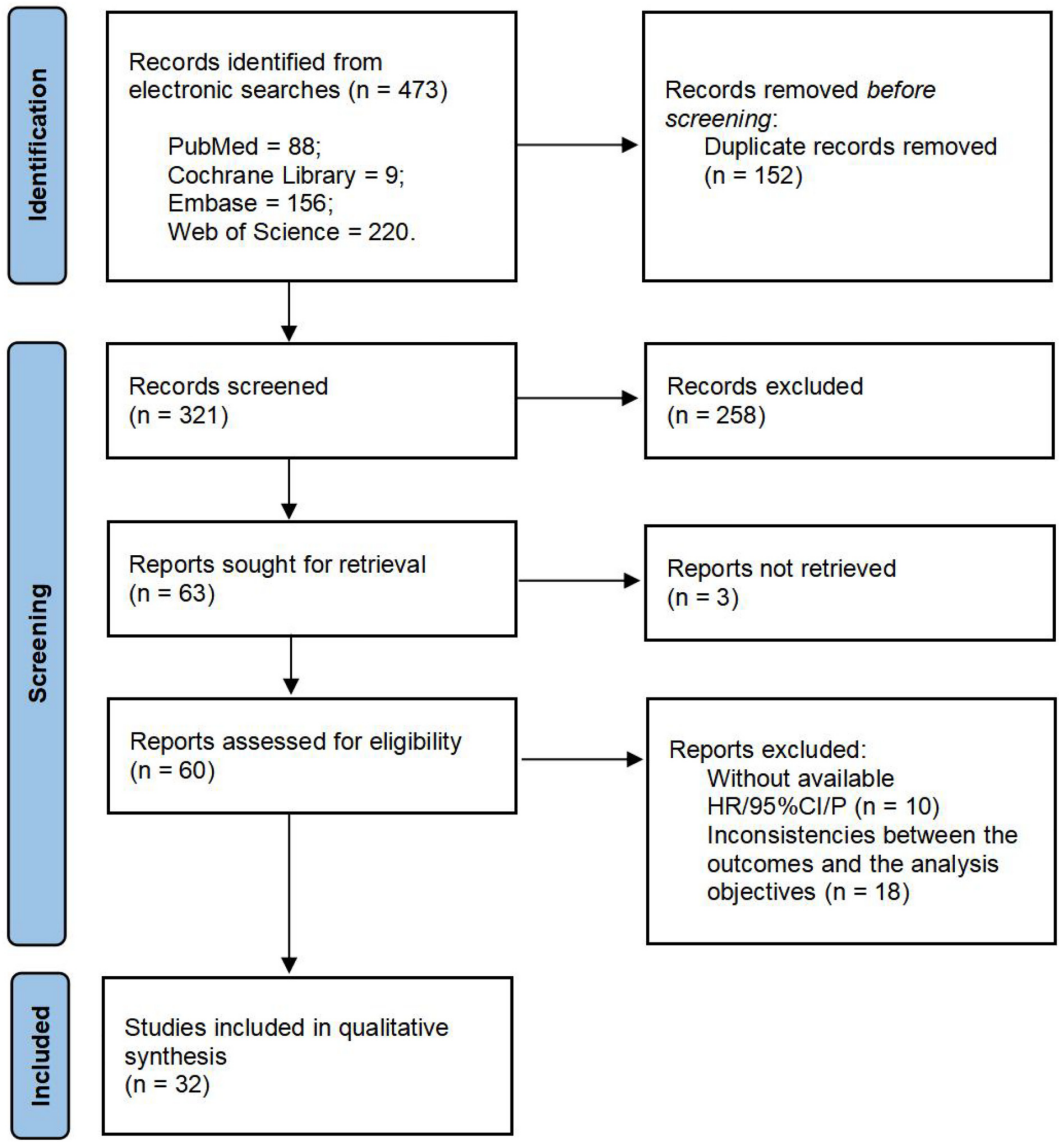
Flow diagram of eligible studies selection.

We conducted a systematic search in PubMed, Embase, Web of Science, and the Cochrane Library databases, initially identifying 473 articles. After removing 152 duplicate records, 321 articles remained. Following the screening of titles and abstracts, 258 studies were excluded due to irrelevant topics, being reviews or meta-analyses, conference abstracts, or conference proceedings. Of the remaining 63 articles, 31 were excluded due to missing data, quality issues, inability to access full text, or inconsistencies between the outcomes and the analysis objectives. Ultimately, 32 studies were included in the meta-analysis, encompassing a total of 10,352 patients. Detailed information on the included studies is provided in Table 1, and the selection process is outlined in Figure 1.

**Table 1:** The main characteristics of included studies.

Year	First Author	Country	Study design	Sample size (female, %)	Tumor type	Surgical procedure	Score	Median/average follow-up	Postoperative chemotherapy, *n*=	Analysis model	Outcome	Study type	Cutoff value
2010	Tadahiro Nozoe (54)	Japan	R	248 (70,28.2%)	Gastric cancer	Gastrectomy	PNI	NA	NA	M	OS	S	49.7
2011	Mitsuro Kanda (55)	Japan	R	268 (102,38.1%)	Pancreatic cancer	Radical surgery for pancreatic cancer	PNI	NA	NA	M	OS	S	45
2016	Mitsuro Kanda (56)	Japan	R	260 (68,26.2%)	Gastric cancer	Gastrectomy	PNI	NA	137	M	OS	S	47
2016	Katsunobu Sakurai (57)	Japan	R	594 (166,27.9%)	Gastric cancer	Gastrectomy	PNI	56 months	NA	M	OS	S	45
2016	Tadafumi Asaoka (58)	Japan	R	46 (24,52.2%)	Pancreatic cancer	Radical surgery for pancreatic cancer	PNI	NA	26	M	OS	S	47
2016	Joji Watanabe (59)	Japan	R	46 (20,43.5%)	Pancreatic cancer	Radical surgery for pancreatic cancer	PNI	NA	30	U	OS	S	40
2017	Ding Peng (60)	China	R	1,360 (408,30%)	Renal cancer	Nephrectomy	PNI	67 months	NA	M	OS	S	47.625
2018	Tatsunori Miyata (31)	Japan	R	71 (26,36.6%)	Liver cancer	Liver resection	CONUT	36.9 months	NA	M	OS	S	2
2018	Norifumi Harimoto (61)	Japan	R	882 (296,33.6%)	Liver cancer	Liver resection	CONUT	NA	NA	M	OS	M	4
2018	Lei Li (62)	China	R	261 (46,17.6%)	Liver cancer	Liver resection	GNRI	NA	NA	U	OS	S	82
2019	Masahide Ikeguchi (63)	Japan	R	50 (18,36%)	Pancreatic cancer	Radical surgery for pancreatic cancer	PNI	NA	NA	M	OS	S	46
2019	Shinichi Ikuta (64)	Japan	R	136 (60,44.1%)	Pancreatic cancer	Radical surgery for pancreatic cancer	PNI	16.8 months	112	U	OS	S	48.8
2019	Shunsuke Onoe (65)	Japan	R	165 (76,46.1%)	Pancreatic cancer	Radical surgery for pancreatic cancer	PNI	1,788 days	66	M	OS	S	38
2019	Satoshi Suzuki (66)	Japan	R	211 (70,33.2%)	Gastric cancer	Gastrectomy	CONUT	47 months	NA	M	OS	S	5
2019	Song Ryo (32)	Japan	R	626 (191,30.5%)	Gastric cancer	Gastrectomy	CONUT	49.2 months	384	M	OS	M	2
2020	Yanwu Sun (67)	China	R	128 (50,39.1%)	Colorectal cancer	Radical resection of colorectal cancer	PNI	43 months	NA	M	OS	S	43.8
2020	Hitomi Takechi (33)	Japan	R	182 (52,28.6%)	Gastric cancer	Gastrectomy	PNI	39 months	33	M	OS	S	45
2020	Sojun Hoshimoto (68)	Japan	R	211 (92,43.6%)	Pancreatic cancer	Radical surgery for pancreatic cancer	PNI	19 months	113	U	OS	S	47.25
2020	Shuai-Shuai Xu (69)	China	R	582 (263,45.2)	Pancreatic cancer	Radical surgery for pancreatic cancer	PNI	NA	477	U	OS	S	53.1
2020	Masahiro Sasahara (70)	Japan	R	842 (259,30.8%)	Gastric cancer	Gastrectomy	PNI	48.6 months	517	U	OS	M	47
2020	Masaru Sasaki (71)	Japan	R	313 (112,35.8%)	Colorectal cancer	Radical resection of colorectal cancer	GNRI	60.5 months	NA	M	OS	S	98
2021	Yongmei Zhu (72)	China	R	196 (78,39.8%)	Colorectal cancer	Radical resection of colorectal cancer	PNI	NA	196	M	OS	S	45.61
2021	Shunsuke Onoe (73)	Japan	R	187 (78,41.7%)	Pancreatic cancer	Radical surgery for pancreatic cancer	PNI	1194 days	147	M	S	S	36
2021	Shinji Itoh (74)	Japan	R	589 (263,44.7%)	Pancreatic cancer	Radical surgery for pancreatic cancer	PNI	NA	NA	M	OS	M	46
2021	Fumihiro Terasaki (75)	Japan	R	307 (125,40.7%)	Pancreatic cancer	Radical surgery for pancreatic cancer	CONUT	NA	NA	M	OS	S	4
2022	Tomoki Ryu (76)	Japan	R	341 (134,39.3%)	Liver cancer	Microwave coagulo-necrotic therapy	PNI	69 months	NA	M	OS	S	44.5
2022	Huifang Dai (77)	China	R	202 (138,68.3%)	Gallbladder cancer	Cholecystectomy	GNRI	NA	NA	M	OS	S	98
2022	Tamuro Hayama (78)	Japan	R	259 (115,44.4%)	Colorectal cancer	Radical resection of colorectal cancer	GNRI	1214 days	NA	M	OS	S	101.1
2023	Siyi Lu (79)	China	R	300 (95,31.7%)	Colorectal cancer	Radical resection of colorectal cancer	CONUT	NA	NA	M	OS	S	5
2023	Shinichi Ikuta (80)	Japan	R	213 (95,44.6%)	Cholangiocarcinoma	Resection of cholangiocarcinoma	GNRI	72.6 months	140	M	OS	S	98
2024	Hironobu Suto (81)	Japan	R	153 (67,43.8%)	Pancreatic cancer	Radical surgery for pancreatic cancer	PNI	44 months	79	M	OS	S	38.6
2024	Daisuke Ogawa (82)	Japan	R	123 (41,33.3%)	Liver cancer	Liver resection	GNRI	37 months	NA	M	OS	S	105

R, retrospective; NA, not applicable; PNI, prognostic nutritional index; CONUT, controlling nutritional status; GNRI, geriatric nutritional risk index; M, multivariate; U, univariate; OS, overall survival; S, single-center; M, multi-center; Score means nutritional status score.

### 3.2 Clinical characteristic of enrolled studies

The main characteristics of the included studies are presented in Table 1. These studies were retrospective in design and primarily published within the past fifteen years. All included studies assessed the preoperative nutritional status of surgical patients using PNI, CONUT, or GNRI. Among them, 20 studies mainly used PNI to assess preoperative nutritional status, 6 studies primarily used the GNRI score to assess preoperative nutritional status, and the remaining 6 studies mainly used the CONUT score to evaluate patients’ preoperative nutritional status. Among the 32 studies, 12 were based on pancreatic cancer surgery populations, 7 on gastric cancer surgery populations, 5 on liver cancer surgery populations, 5 on colorectal cancer surgery populations, and 1 each on kidney cancer, gallbladder cancer, and cholangiocarcinoma surgery populations. Of the 32 studies, 25 were from Japan, and 7 were from China. Twenty-eight studies were single-center, while 4 were multi-center. The sample sizes of the included studies ranged from 46 to 1,360, with 20 studies having a sample size of 200 or more. All included studies investigated the correlation between preoperative nutritional status and OS. Multivariate analysis was performed in 26 out of the 32 studies. The NOS scores ranged from 6 to 8, as shown in Supplementary Table S1.

### 3.3 Relationship between preoperative nutritional status and OS

**Figure 2:**
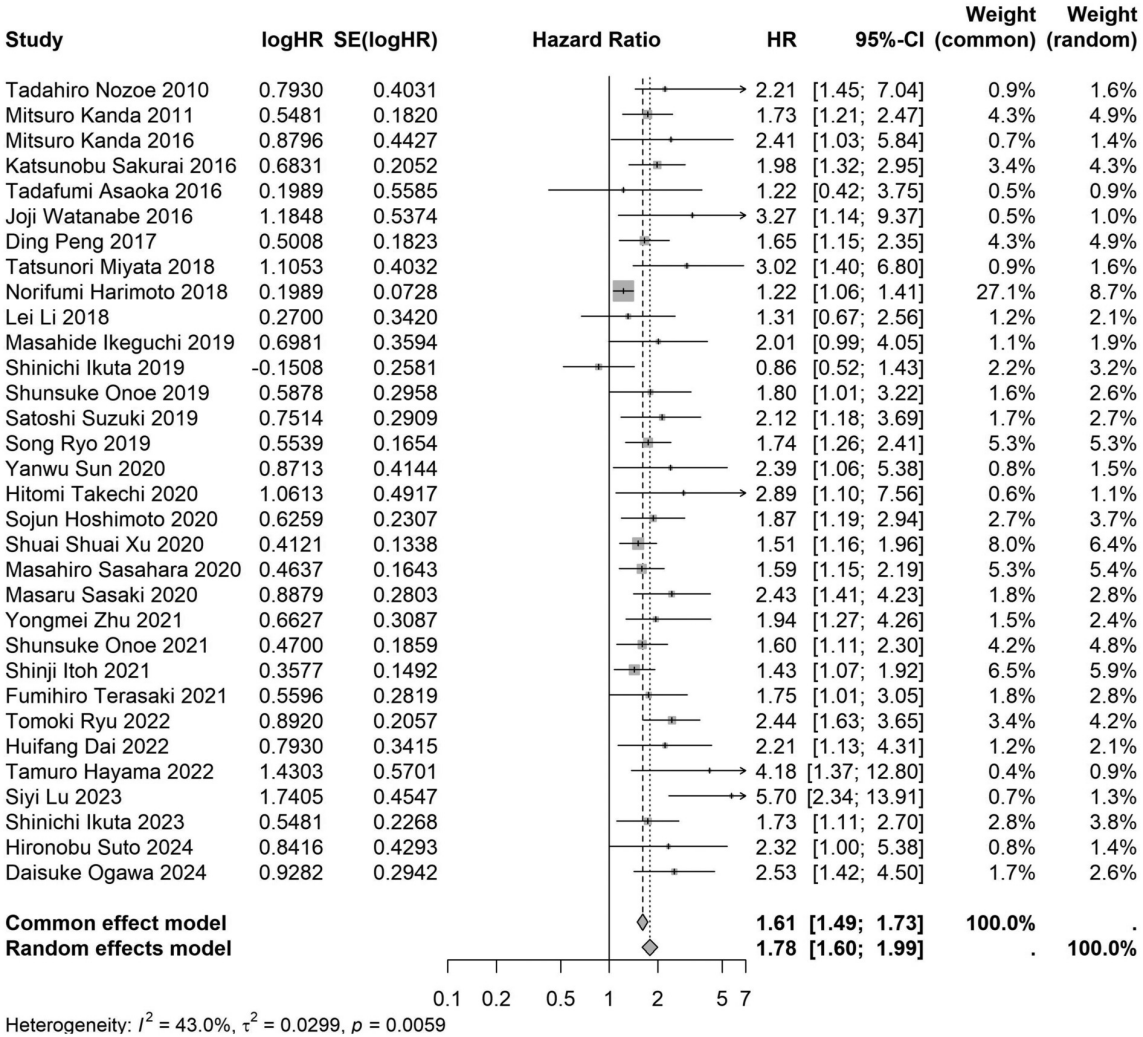
Forest plot of the association between preoperative nutritional status and OS. (OS, overall survival; HR, hazard ratio; Cl, confidence interval).

**Figure 3:**
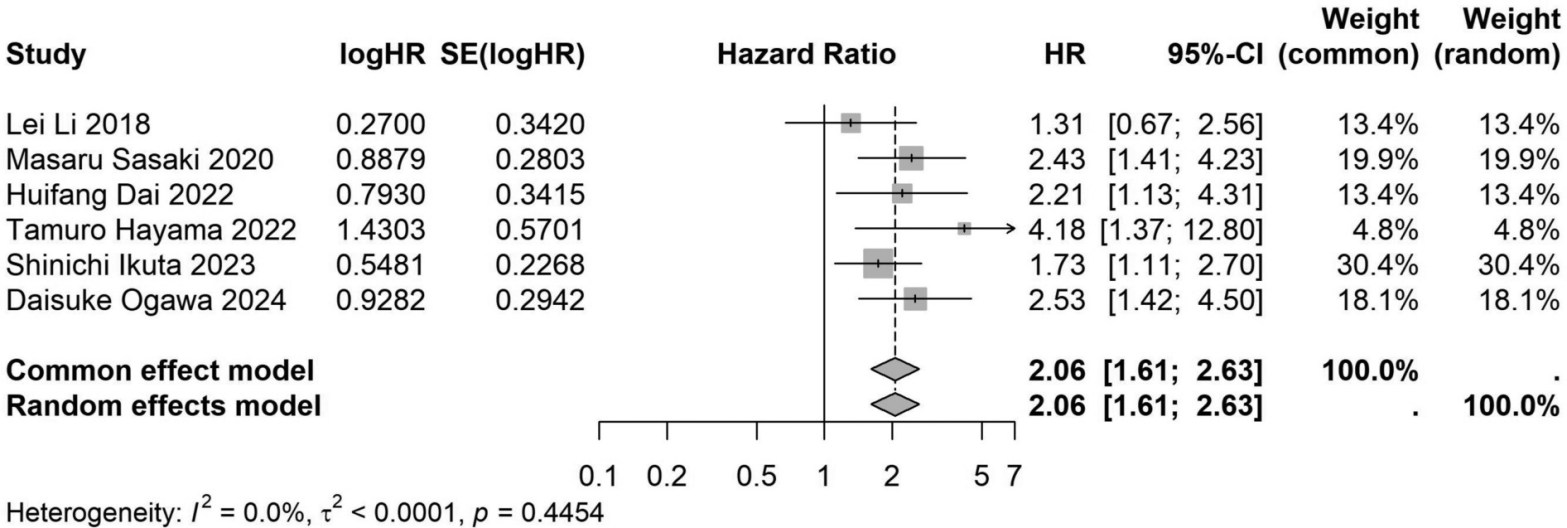
Forest plot of the association between preoperative GNRI and OS. (GNRI, geriatric nutritional risk index; OS, overall survival; HR, hazard ratio; Cl, confidence interval).

**Figure 4:**
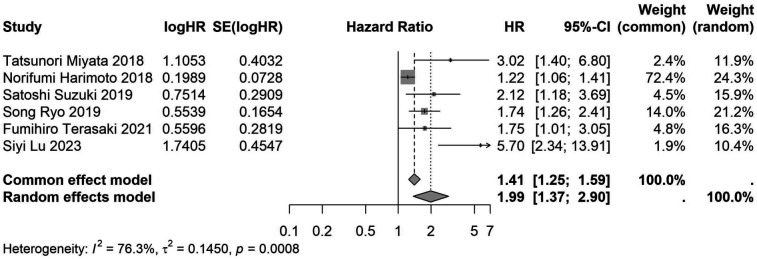
Forest plot of the association between preoperative CONUT and OS. (CONUT, controlling nutritional status; OS, overall survival; HR, hazard ratio; Cl, confidence interval).

**Figure 5:**
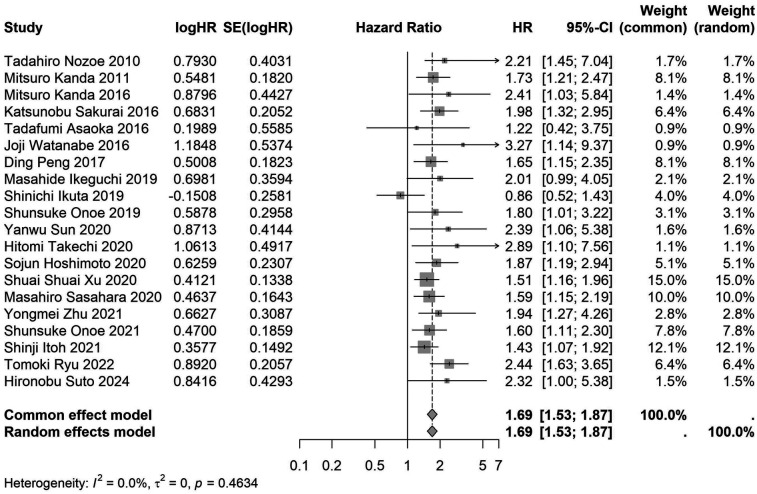
Forest plot of the association between preoperative PNI and OS. (PNI, prognostic nutritional index; OS, overall survival; HR, hazard ratio; Cl, confidence interval).

**Figure 6:**
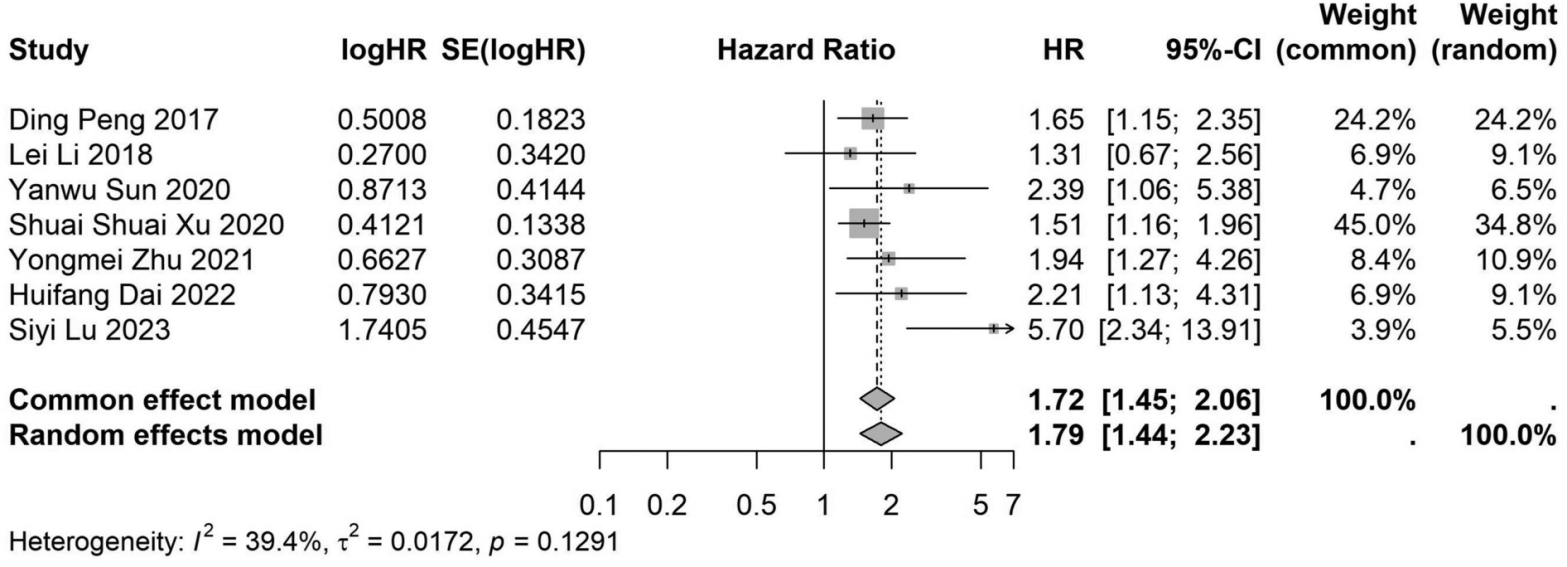
Forest plot of the association between preoperative nutritional status and OS based on the Chinese study. (OS, overall survival; HR, hazard ratio; Cl, confidence interval).

**Figure 7:**
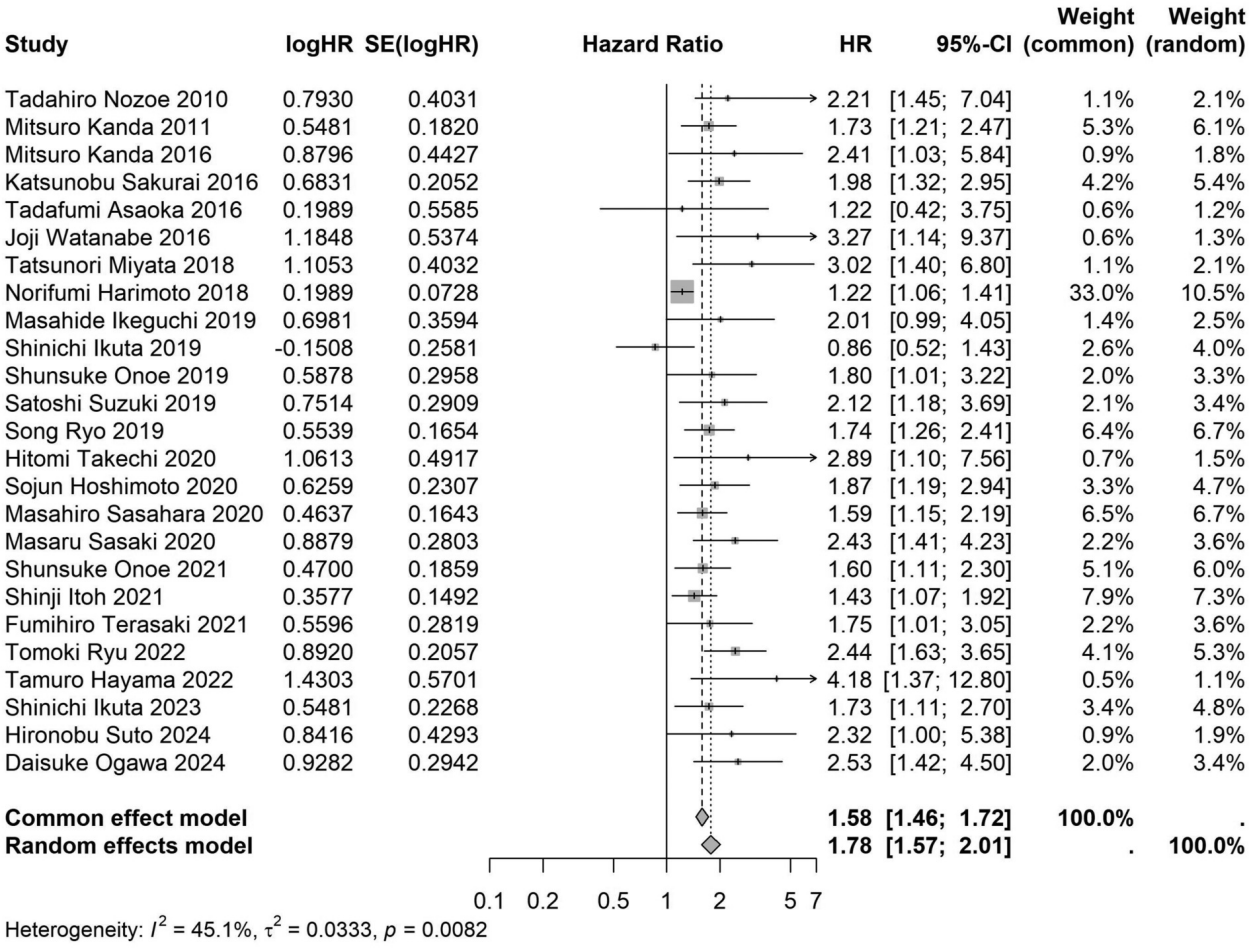
Forest plot of the association between preoperative nutritional status and OS based on the Japanese study. (OS, overall survival; HR, hazard ratio; Cl, confidence interval).

**Figure 8:**
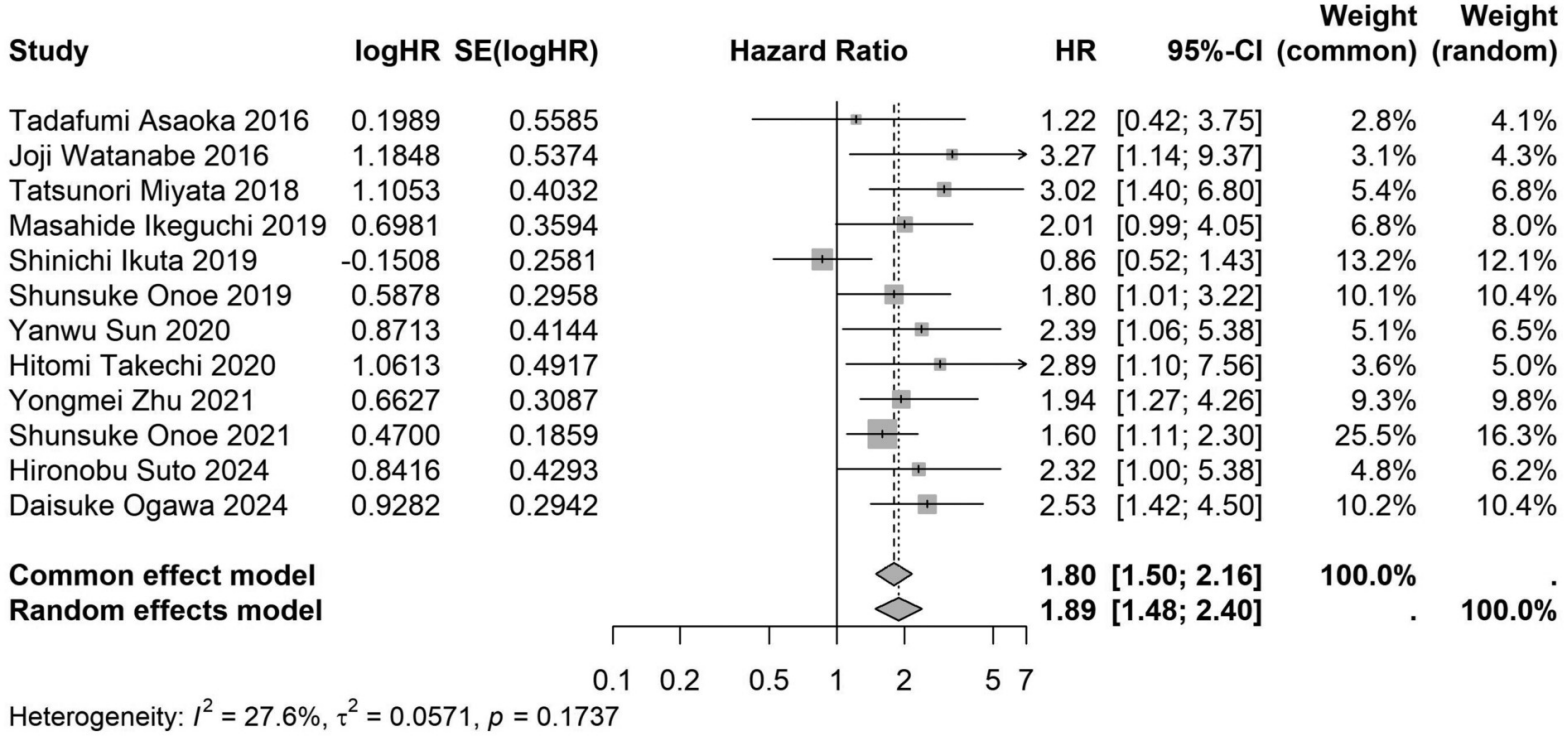
Forest plot of the association between preoperative nutritional status and OS based on the study with a sample size <200. (OS, overall survival; HR, hazard ratio; Cl, confidence interval).

**Figure 9:**
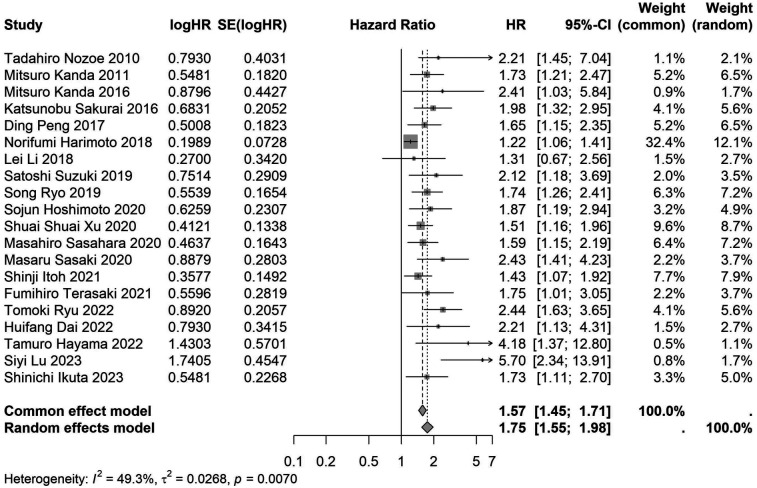
Forest plot of the association between preoperative nutritional status and OS based on the study with a sample size ≥200. (OS, overall survival; HR, hazard ratio; Cl, confidence interval).

**Figure 10:**
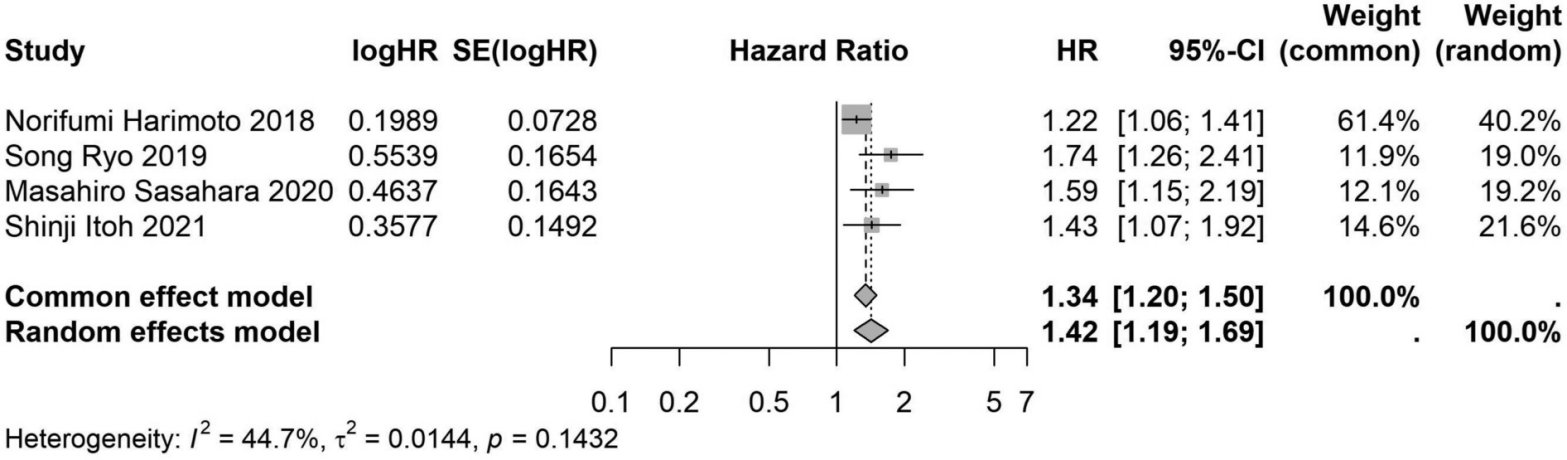
Forest plot of the association between preoperative nutritional status and OS based on the study multicenter design. (OS, overall survival; HR, hazard ratio; Cl, confidence interval).

**Figure 11:**
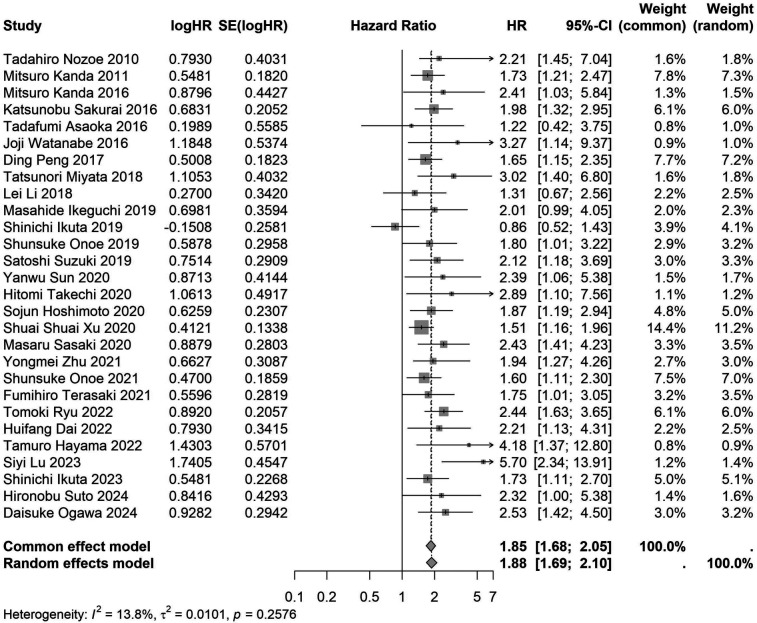
Forest plot of the association between preoperative nutritional status and OS based on the study single-center design. (OS, overall survival; HR, hazard ratio; Cl, confidence interval).

**Figure 12:**
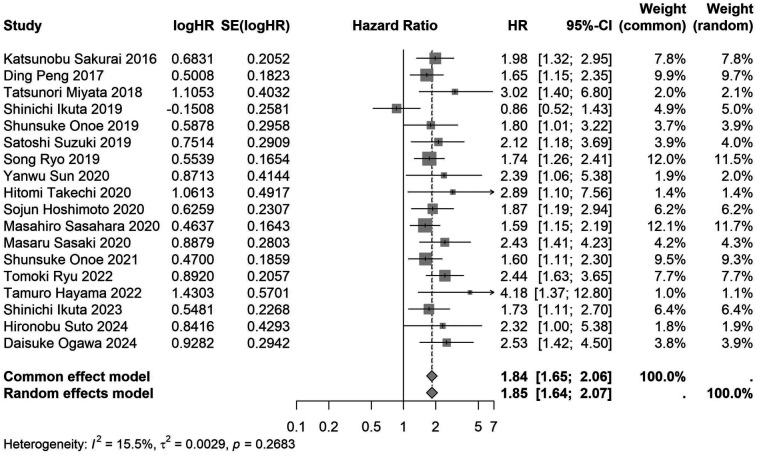
Forest plot of the association between preoperative nutritional status and OS based on the study that clearly documented median or mean follow-up durations. (OS, overall survival; HR, hazard ratio; Cl, confidence interval).

**Figure 13:**
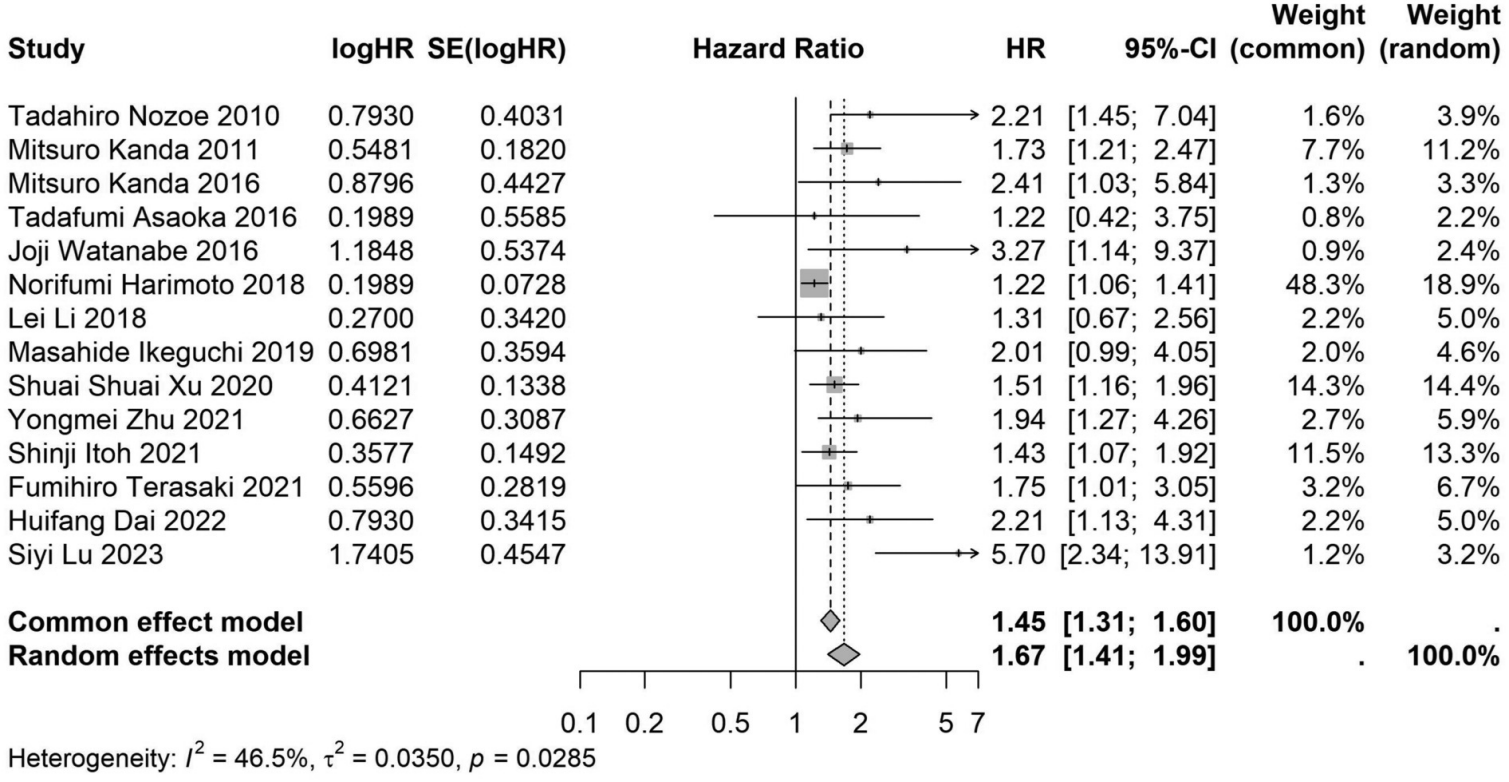
Forest plot of the association between preoperative nutritional status and OS based on the study that did not clearly documented median or mean follow-up durations. (OS, overall survival; HR, hazard ratio; Cl, confidence interval).

**Figure 14:**
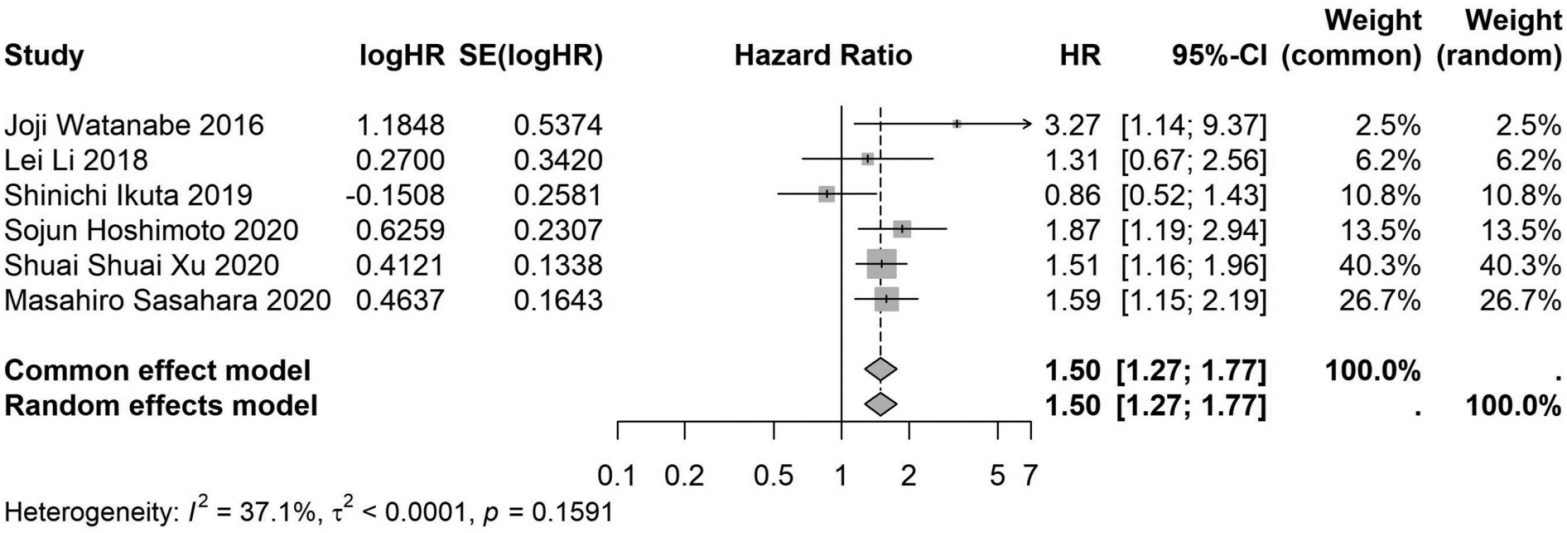
Forest plot of the association between preoperative nutritional status and OS based on the study using univariate analytical model. (OS, overall survival; HR, hazard ratio; Cl, confidence interval).

**Figure 15:**
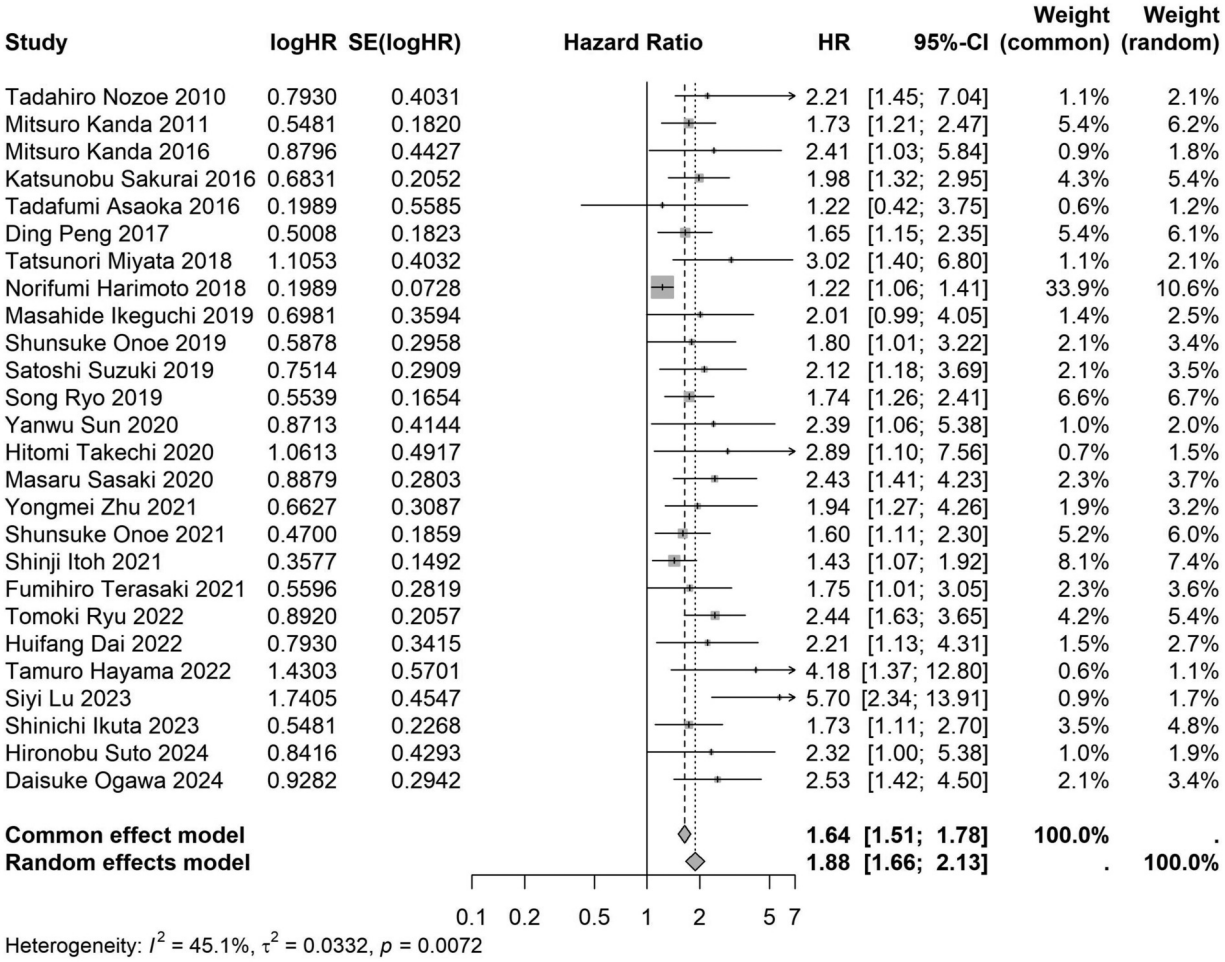
Forest plot of the association between preoperative nutritional status and OS based on the study using multivariate analytical model. (OS, overall survival; HR, hazard ratio; Cl, confidence interval).

**Figure 16:**
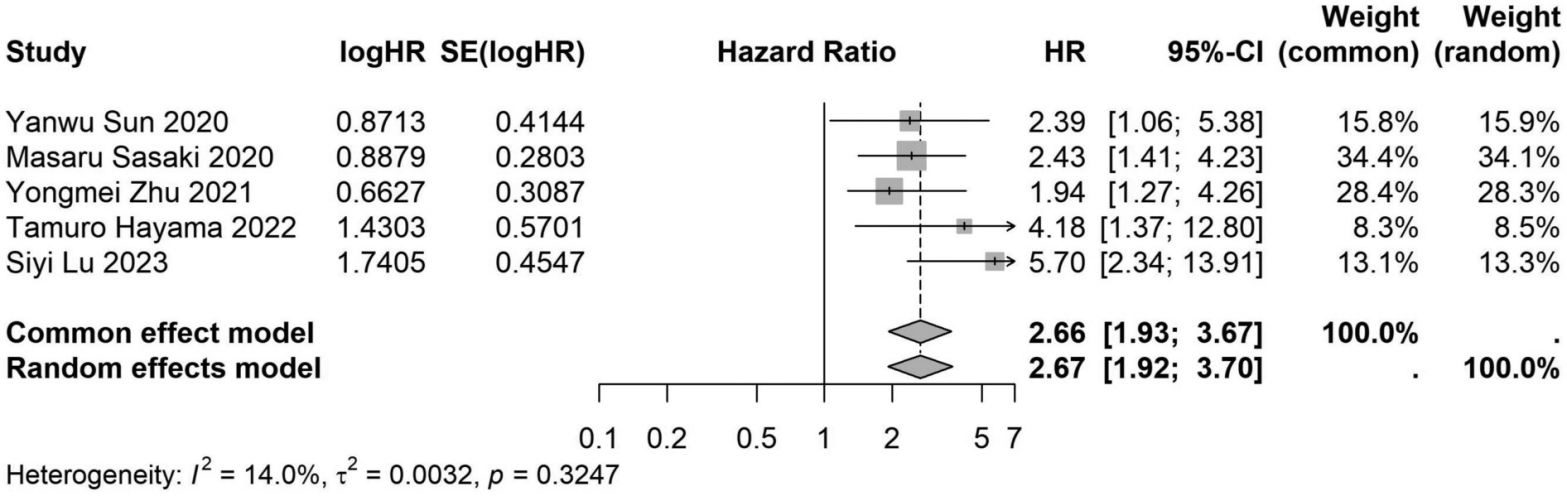
Forest plot of the association between preoperative nutritional status and OS based on the study focus on colorectal cancer. (OS, overall survival; HR, hazard ratio; Cl, confidence interval).

**Figure 17:**
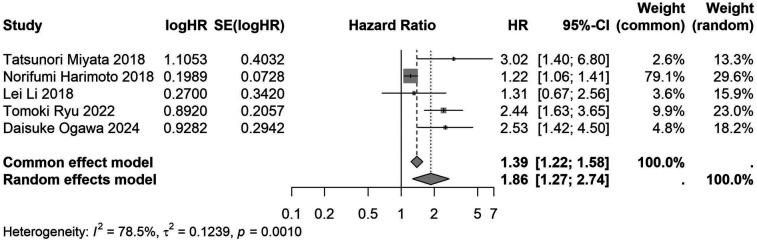
Forest plot of the association between preoperative nutritional status and OS based on the study focus on liver cancer. (OS, overall survival; HR, hazard ratio; Cl, confidence interval).

**Figure 18:**
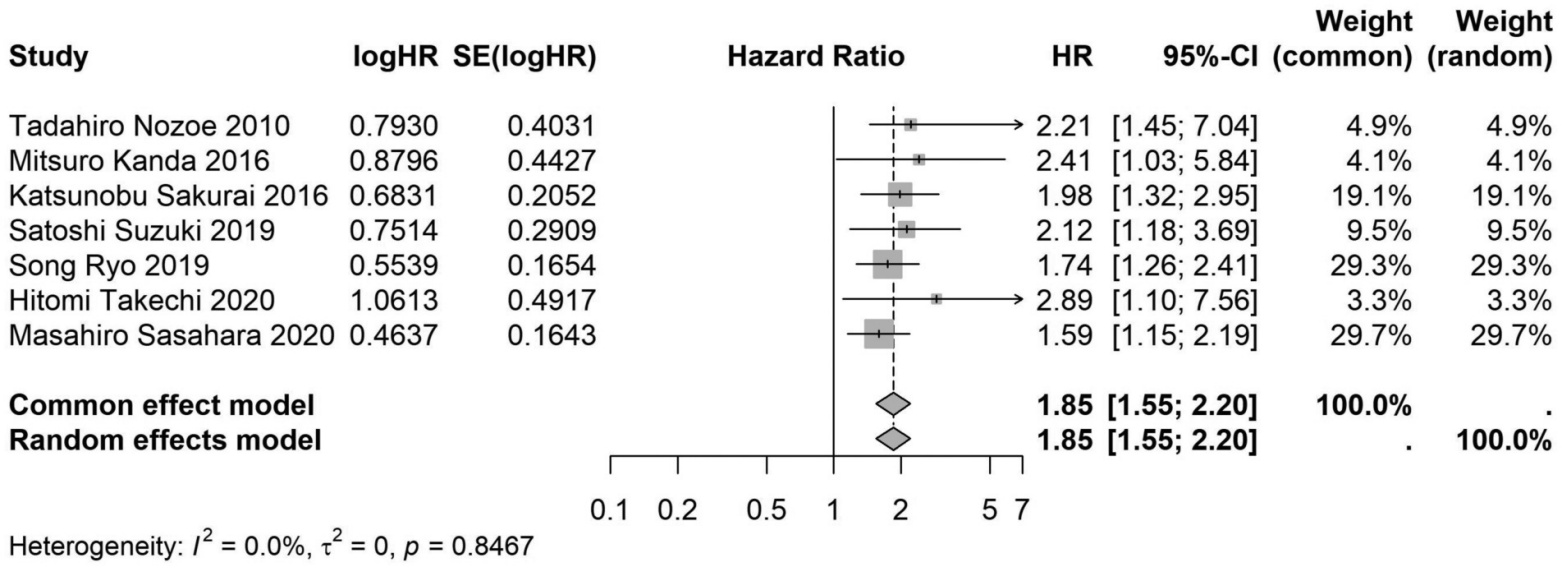
Forest plot of the association between preoperative nutritional status and OS based on the study focus on gastric cancer. (OS, overall survival; HR, hazard ratio; Cl, confidence interval).

**Figure 19:**
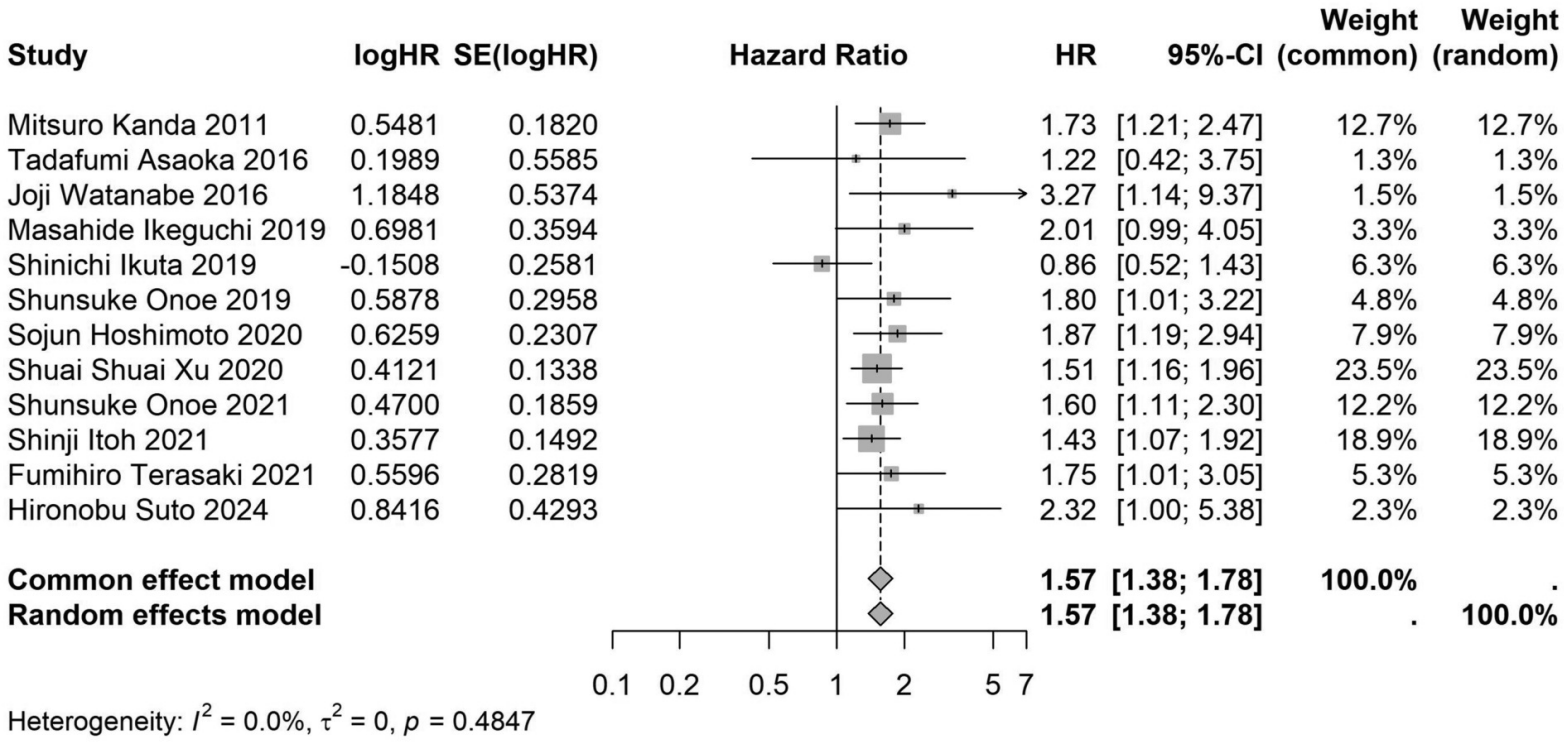
Forest plot of the association between preoperative nutritional status and OS based on the study focus on pancreatic cancer. (OS, overall survival; HR, hazard ratio; Cl, confidence interval).

A total of 32 studies, including 10,352 patients, investigated the relationship between preoperative nutritional status and OS. The combined forest plot demonstrated that preoperative malnutrition was associated with poorer OS in patients with abdominal cancer surgery (HR = 1.61, 95% CI 1.49–1.73, *p* < 0.001) (Figure 2). Subgroup analyses based on methods of nutritional status assessment, country, sample size, study design, follow-up duration, analytical models, and cancer types revealed consistent results (Table 2). Specifically, whether assessed using the GNRI score (HR = 2.06, 95% CI 1.21–2.63, *p* < 0.001), CONUT score (HR = 1.99, 95% CI 1.37–2.90, *p* < 0.001), or PNI score (HR = 1.69, 95% CI 1.53–1.87, *p* < 0.001), preoperative malnutrition was consistently associated with worse OS in patients (Figures 3–5). Studies from China (HR = 2.13, 95% CI 1.50–3.02, *p* < 0.001) and Japan (HR = 1.58, 95% CI 1.46–1.72, *p* < 0.001) both supported this association between preoperative malnutrition and poorer OS (Figures 6 and 7). Furthermore, studies with a sample size of less than 200 (HR = 1.80, 95% CI 1.50–2.16, *p* < 0.001) and those with a sample size of 200 or more (HR = 1.84, 95% CI 1.60–2.12, *p* < 0.001) also demonstrated that preoperative malnutrition was linked to worse OS (Figures 8, 9). Both multicenter (HR = 1.34, 95% CI 1.20–1.50, *p* < 0.001) and single-center studies (HR = 1.85, 95% CI 1.68–2.05, *p* < 0.001) yielded similar findings (Figures 10, 11). Studies that clearly documented median or mean follow-up durations (HR = 1.84, 95% CI 1.65–2.06, *p* < 0.001) and those that did not (HR = 1.45, 95% CI 1.31–1.60, *p* < 0.001) also showed comparable results (Figures 12, 13). Both univariate analysis (HR = 1.50, 95% CI 1.27–1.77, *p* < 0.001) and multivariate analysis (HR = 1.64, 95% CI 1.51–1.78, *p* < 0.001) studies confirmed the same association (Figures 14, 15). After categorizing by tumor type, subgroup analyses for colorectal cancer (HR = 2.66, 95% CI 1.93–3.67, *p* < 0.001), liver cancer (HR = 1.86, 95% CI 1.27–2.74, *p* = 0.001), gastric cancer (HR = 1.85, 95% CI 1.55–2.20, *p* < 0.001), and pancreatic cancer (HR = 1.57, 95% CI 1.38–1.78, *p* < 0.001) also demonstrated that preoperative malnutrition was associated with poorer OS, as shown in Figures 16–19. In addition, studies focus on cholangiocarcinoma (HR = 1.73, 95% CI 1.11–1.2.70, *p* < 0.001), gallbladder cancer (HR = 2.21, 95% CI 1.13–4.31, *p* = 0.020), and renal cancer (HR = 1.65, 95% CI 1.15–2.35, *p* = 0.006) each demonstrated the correlation between preoperative malnutrition and poorer OS (Supplementary Figures S1–S3).

**Table 2:** The results of subgroup analyses and meta-regression analyses.

Subgroup	No. study	No. patients	Random-effects model		Fixed-effects model		Heterogeneity		*p*-value (meta-reg)
			HR (95%, CI)	*p*-value	HR (95%, CI)	*p*-value	*p*-value (heterogeneity)	*I*^2^ (%)	
Methods of nutritional status assessment									
GNRI	6	1,371	2.06 (1.61–2.63)	<0.001	2.06 (1.61–2.63)	<0.001	0.445	0	0.513
CONUT	6	2,397	1.99 (1.37–2.90)	<0.001	1.41 (1.25–1.59)	<0.001	<0.001	76.3	–
PNI	20	6,584	1.69 (1.53–1.87)	<0.001	1.69 (1.53–1.87)	<0.001	0.463	0	–
Country									
China	7	3,029	1.79 (1.44–2.23)	<0.001	1.72 (1.45–2.06)	<0.001	0.129	39.4	0.817
Japan	25	7,323	1.78 (1.57–2.01)	<0.001	1.58 (1.46–1.72)	<0.001	0.008	45.1	–
Sample size									
<200	12	1,483	1.89 (1.48–2.40)	<0.001	1.80 (1.50–2.16)	<0.001	0.173	27.6	0.680
≥200	20	8,869	1.75 (1.55–1.98)	<0.001	1.57 (1.45–1.71)	<0.001	0.007	49.2	–
Study design									
Multiple center	4	2,939	1.42 (1.19–1.69)	<0.001	1.34 (1.20–1.50)	<0.001	0.143	44.7	0.005
Single center	28	7,413	1.88 (1.69–2.10)	<0.001	1.85 (1.68–2.05)	<0.001	0.258	13.8	–
Follow-up duration									
Without a clearly defined follow-up time	14	4,237	1.45 (1.31–1.61)	<0.001	1.67 (1.41–1.99)	<0.001	0.028	46.5	0.188
With a clearly defined follow-up time	18	6,115	1.85 (1.64–2.07)	<0.001	1.84 (1.65–2.06)	<0.001	0.268	15.5	–
Analytical model									
Univariate	6	2,078	1.50 (1.27–1.77)	<0.001	1.50 (1.27–1.77)	<0.001	0.159	37.1	0.090
Multivariate	26	8,274	1.64 (1.51–1.78)	<0.001	1.88 (1.66–2.13)	<0.001	0.007	45.1	–
Tumor type									
Colorectal cancer	5	1,196	2.67 (1.92–3.70)	<0.001	2.66 (1.93–3.67)	<0.001	0.325	14.0	0.067
Liver cancer	5	1,678	1.86 (1.27–2.74)	0.001	1.39 (1.22–1.58)	<0.001	<0.001	78.5	–
Gastric cancer	7	2,963	1.85 (1.55–2.20)	<0.001	1.85 (1.55–2.20)	<0.001	0.847	0	–
Pancreatic cancer	12	2,740	1.57 (1.38–1.78)	<0.001	1.57 (1.38–1.78)	<0.001	0.485	0	–

### 3.4 Meta-regression

The meta-regression analysis revealed that, with the exception of the subgroup analysis based on study design (single-center vs. multi-center studies) (*p* = 0.005), no significant heterogeneity was observed in the other subgroup analyses, suggesting that our results are robust (Table 2). Furthermore, these findings imply that the type of study design (single-center or multi-center) may contribute to the observed heterogeneity.

### 3.5 Sensitivity analysis and publication bias

**Figure 20:**
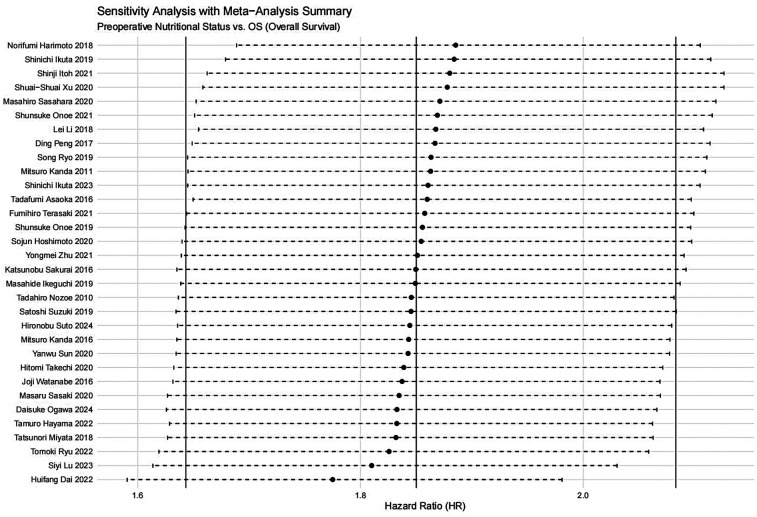
Sensitivity analysis for the association between preoperative nutritional status and OS (OS, overall survival).

**Figure 21:**
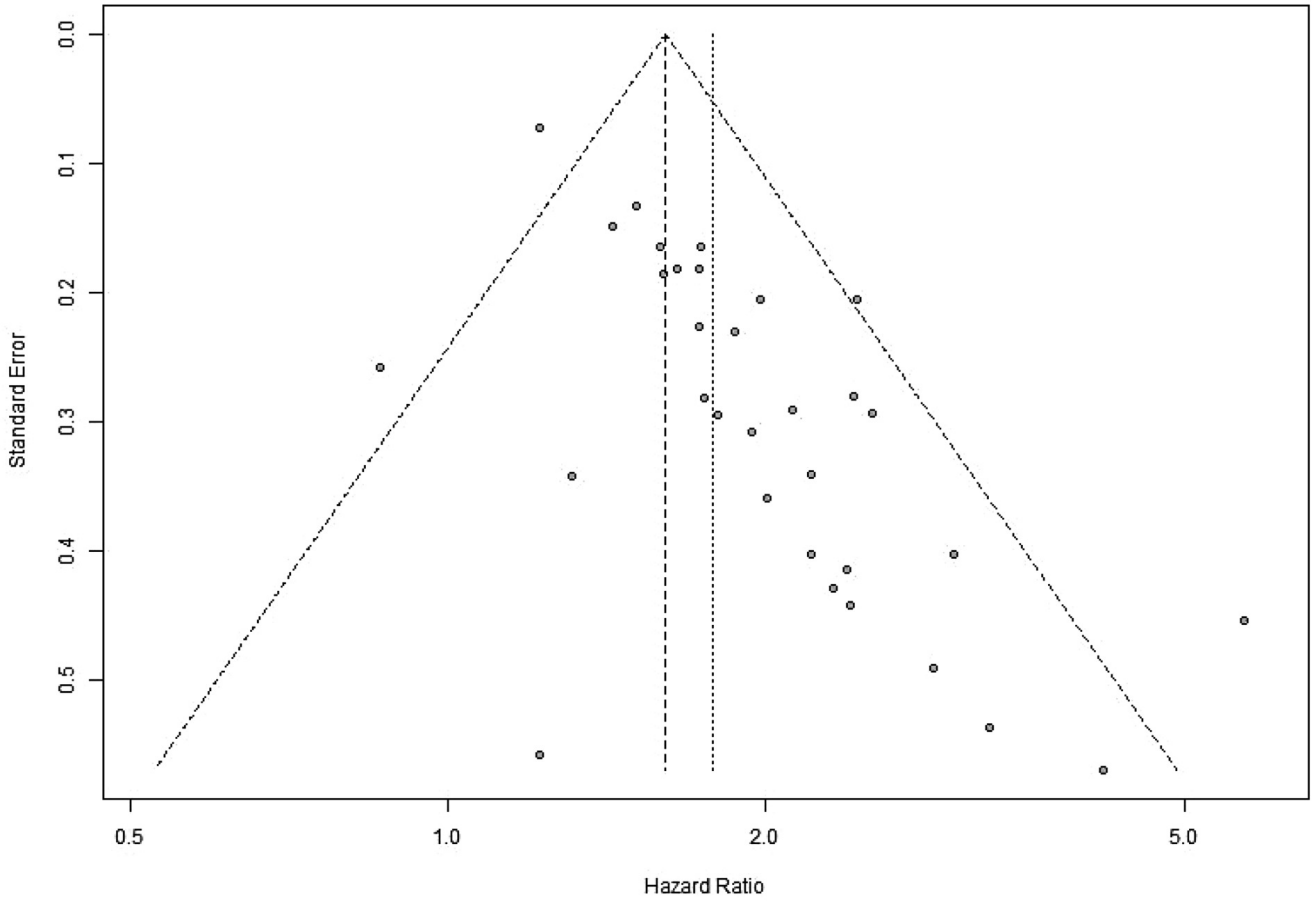
Funnel plot for evaluation of publication bias or small-study effects in OS (OS, overall survival).

A sensitivity analysis was performed to evaluate the impact of individual studies on the pooled HR for OS. The results indicated that excluding any single study did not significantly affect the pooled HR (Figure 20). Additionally, publication bias was assessed, and visual inspection of the funnel plot revealed no apparent asymmetry (Figure 21).

## 4 Discussion

Our systematic review and meta-analysis indicate that preoperative nutritional status is a critical prognostic factor for OS following abdominal tumor surgery. Several studies involving colorectal cancer, gastric cancer, pancreatic cancer, and hepatocellular carcinoma have shown that poor nutritional status, characterized by low serum albumin levels, decreased PNI, reduced GNRI, elevated CONUT, reduced skeletal muscle mass, and adverse inflammation-nutrition composite scores, is associated with lower long-term survival rates and increased postoperative complications (30–35). These findings underscore the necessity of incorporating routine and standardized nutritional assessments into the preoperative evaluation of patients with abdominal tumors. Over the past few decades, advances in surgical techniques and perioperative care have further emphasized the need to reassess the relationship between nutrition and clinical outcomes. In the era of ERAS, optimizing patients’ preoperative physiological status has gained increasing importance. ERAS protocols advocate for early mobilization, minimally invasive surgical techniques, and targeted perioperative nutritional support as key strategies to reduce postoperative complications and improve recovery times (36–38). Recent studies have demonstrated that adequate nutritional intervention can mitigate the harmful impact of preoperative malnutrition on OS (39), highlighting the potential positive effects of improving preoperative nutritional status on patient prognosis.

Additionally, with the promotion of precision medicine and the implementation of individualized treatment models, there is a growing demand for comprehensive assessments of patients’ overall condition, including their nutritional and functional status. Clarifying the prognostic role of preoperative nutritional status in abdominal tumor surgery not only provides risk stratification and decision-making support for surgeons but also plays a pivotal role in multidisciplinary clinical pathways. This is particularly relevant for elderly patients and high-risk groups, where optimizing preoperative nutritional status can greatly enhance postoperative recovery, improve overall survival, and quality of life. Such improvements can profoundly impact the development of modern surgical treatment models, transitioning toward an integrated approach of “prevention-intervention-recovery” (40).

Malnutrition often leads to increased postoperative complication rates, prolonged hospital stays, and heightened mortality risk (40). In addition to these adverse outcomes, malnourished patients are more prone to infections, exhibit poorer responses to cancer treatments, and experience impaired overall prognosis (30, 41). Previous studies have indicated that nutrition plays a critical role in immune response, with malnutrition leading to immune deficiencies (42). Furthermore, malnutrition is a major cause of zinc deficiency, which impairs cell-mediated immune function and is associated with host cell-mediated immune damage (43). Beyond impairing host immunity, poor nutritional status can alter drug metabolism, reduce tolerance to adjuvant therapies, and diminish patients’ overall functional reserves (44). In summary, maintaining optimal preoperative nutritional status is crucial for the recovery of patients undergoing tumor surgery.

While there are existing studies investigating the prognostic impact of individual preoperative nutritional assessments such as PNI, GNRI, or CONUT scores on tumor surgery outcomes, there is still a lack of integrative evaluations on the combined application of these scoring systems, particularly in terms of their applicability and effectiveness across different tumor types. Therefore, we conducted this meta-analysis by integrating studies that included these three nutritional scoring systems and abdominal tumors. This approach enhances the evidential value of the conclusions. To the best of our knowledge, our study is the first meta-analysis to combine multiple simple, objective nutritional scores with various types of abdominal tumors.

Ultimately, this study included a total of 32 studies and 10,352 patients who underwent abdominal cancer surgery. Our findings indicate that preoperative malnutrition negatively impacts the OS of patients. To assess the heterogeneity between the included studies and the influence of different study characteristics on the prognostic value of preoperative nutritional status scoring, we performed subgroup and meta-regression analyses based on the preoperative nutritional status assessment methods, the country of origin of the study, sample size, study design, follow-up duration, data analysis methods, and tumor type. We found that, regardless of these factors, preoperative malnutrition was consistently associated with significantly poorer OS. Sensitivity analyses further confirmed the robustness of our results. This suggests that preoperative nutritional status may serve as an important prognostic indicator for patients undergoing abdominal tumor surgery.

The PNI is calculated using serum albumin levels and peripheral blood lymphocyte count to assess the immune-nutritional status and a low PNI indicates poor prognosis (22); The CONUT score is calculated based on serum albumin levels, total lymphocyte count, and total cholesterol levels, with higher scores indicating worse nutritional statu (23). The GNRI is calculated using serum albumin concentration and the ratio of actual body weight to ideal body weight, and it is also used to evaluate nutritional risk, with lower values indicating higher risk of adverse events (24). All three indices are key tools for evaluating patients’ nutritional status and prognosis. These measures, based on specific laboratory markers, offer higher reliability compared to many subjective scales or imaging markers. These measures, based on specific laboratory markers, offer higher reliability compared to many subjective scales or imaging markers. They are also simpler and more practical for clinical use. The objective nature of these indices, when applied widely in clinical research and practice, can assist physicians in developing more targeted treatment strategies, thus improving overall health outcomes and quality of life for patients. Effective utilization of these scoring systems allows medical teams to better identify high-risk patients, enabling timely intervention to reduce complications and improve survival rates.

Of course, there are certain limitations to our study. For instance, subgroup analyses based on study design (single-center vs. multi-center studies) revealed some heterogeneity, highlighting the need for further investigation to address this potential source of variability in future studies. Additionally, careful and rigorous consideration of research designs related to preoperative nutritional status and prognosis is required. Another limitation is the variability in the definition of “abdominal tumors.” Although our systematic review attempted to encompass a broad range of abdominal tumors, the majority of studies focused on gastric, liver, pancreatic, and colorectal cancers, with relatively few studies on gallbladder and bile duct cancers, as well as renal cancer. This limitation emphasizes the need for future research to include a broader spectrum of abdominal tumors and to analyze outcomes based on tumor type, which may reveal more subtle prognostic differences.

One important factor to consider is the potential cultural or geographical bias inherent in the studies included in our analysis. The majority of the included studies originated from Japan and China, and these regions may have distinct healthcare systems, nutritional practices, and patient demographics compared to Western populations. These geographical differences could influence the generalizability of our findings, as nutritional status assessments, treatment protocols, and patient care approaches may differ across regions. In particular, the prevalence of certain abdominal cancers, as well as the approach to preoperative nutritional interventions, may vary significantly between Eastern and Western countries (45).

While the current meta-analysis provides valuable insights into the impact of preoperative nutritional status on postoperative outcomes in abdominal cancer surgery, it is essential to consider how these findings can be applied to Western populations or global practices. In Western countries, the prevalence of abdominal cancers such as colorectal and liver cancer may differ, and the typical patient demographic may also exhibit different nutritional challenges, such as varying rates of obesity or malnutrition (46, 47). Moreover, the healthcare infrastructure and access to preoperative nutritional interventions may differ, potentially influencing the effectiveness of these interventions.

Future studies in Western or other diverse populations are needed to confirm whether the results from this analysis are consistent across different geographic and cultural settings. It will be important to examine how nutritional assessment tools like PNI, CONUT, and GNRI are applied in Western healthcare systems, as well as to explore the role of different treatment regimens, tumor types, and preoperative interventions in these settings. Such studies could provide a more global perspective on the role of nutrition in cancer surgery and help standardize clinical practices worldwide.

By addressing these cultural and geographical variations, future research will be better equipped to tailor preoperative nutritional strategies for diverse patient populations and enhance the global applicability of these findings.

In addition, heterogeneity in defining malnutrition using various nutritional assessment tools such as PNI, CONUT, and GNRI cutoffs may have influenced the pooled estimates. Future studies should aim to standardize these thresholds to minimize this variability. Although the overall heterogeneity in our study was moderate to low, the CONUT subgroup exhibited substantial heterogeneity (*I*² = 76.3%). This finding may be attributed to the differences in the standardization of the CONUT score across studies (48). The CONUT score, as a tool for assessing nutritional status, may have been applied differently in various studies, influenced by patient characteristics, tumor types, and treatment protocols. For example, different studies may have used varying CONUT score thresholds or assessment methods, which could be affected by factors such as tumor staging and treatment regimens (49). Certain tumor types, such as gastric, liver, and pancreatic cancers, may show a higher sensitivity to the CONUT score, while other cancers may present different results (50). These factors likely explain the observed heterogeneity in the CONUT subgroup. Future studies should aim to standardize the use of the CONUT score, particularly in multi-center studies involving diverse tumor types and treatment approaches, to reduce such heterogeneity.

Moreover, while study design differences account for some of the heterogeneity, other potential confounders remain unexplained, including tumor stage, neoadjuvant therapies, the complexity of the surgical procedures, and the missing data related to these factors. These factors are likely to contribute to residual heterogeneity and should be carefully considered in future research.

Despite these limitations, our study holds significant clinical value. Integrating nutritional status assessment into preoperative evaluations can help identify patients at risk of reduced survival. This understanding provides a window for preoperative nutritional interventions, which can optimize patient outcomes through tailored dietary counseling, oral nutritional supplementation, and even prehabilitation programs such as resistance training to improve muscle mass. These interventions may be especially important for elderly or frail populations, who typically have limited physiological reserves.

Looking ahead, future research should focus on several key areas. First, large-scale, prospective trials are needed to examine the impact of targeted nutritional interventions on short- and long-term outcomes in abdominal cancer surgery. Standardized nutritional assessment methods (such as PNI, CONUT, and GNRI scores) should be used in studies to facilitate data aggregation and improve the reliability of meta-analytic estimates. Second, future studies should explore the combined effects of nutritional and inflammatory biomarkers, as their interaction appears to play a critical role in tumor progression and postoperative recovery (51).

Moreover, research should explore the integration of nutritional optimization into multimodal prehabilitation programs, which may include physical training, psychosocial support, and medical optimization. Such comprehensive programs can not only improve nutritional status but also enhance patients’ overall psychological resilience, leading to better surgical outcomes (52). Finally, integrating genomic and metabolomic research with nutritional assessment could identify new biomarkers, further refining our understanding of the relationship between host nutritional status and cancer outcomes (53). These interdisciplinary approaches may pave the way for personalized nutritional therapies in oncologic surgery.

In conclusion, our meta-analysis confirms that preoperative nutritional status is one of the key determinants of OS in patients undergoing abdominal cancer surgery. The significant association between poor nutritional indicators and adverse postoperative outcomes underscores the necessity for comprehensive preoperative assessments and the potential for nutritional interventions to improve long-term survival. Despite some heterogeneity and research limitations, a large body of evidence from various cancer types and clinical settings supports the inclusion of nutritional assessments in routine surgical planning and the development of targeted preoperative optimization strategies. Future research, particularly prospective multi-center studies, should standardize assessment tools and develop evidence-based nutritional intervention protocols that can be seamlessly integrated into clinical practice. Ultimately, addressing preoperative nutritional deficiencies may significantly improve surgical outcomes and increase the OS rate of patients with abdominal malignancies.

## Data Availability

The original contributions presented in the study are included in the article/Supplementary Material, further inquiries can be directed to the corresponding author.
