# Supplementary material for: Preoperative nutritional status as a predictor of postoperative overall survival in abdominal tumor surgery: a systematic review and meta-analysis
Source: Front Surg. 2025 Aug 18;12:1645392. doi: 10.3389/fsurg.2025.1645392 (PMC12399654; doi:10.3389/fsurg.2025.1645392)
Supplement: Supplementary file 1 [file Supplementaryfile1.docx]

**Pubmed:**

(nutritional status[MeSH Terms] OR malnutrition[MeSH Terms])

AND

(preoperative malnutrition[Title/Abstract] OR preoperative dystrophy[Title/Abstract] OR preoperative GNRI[Title/Abstract] OR preoperative PNI[Title/Abstract] OR preoperative CONUT[Title/Abstract])

AND

prognosis[Title/Abstract]

**Embase:**

('nutritional status'/exp OR 'malnutrition'/exp)

AND

('preoperative malnutrition' OR 'preoperative dystrophy' OR 'preoperative GNRI' OR 'preoperative PNI' OR 'preoperative CONUT')

AND

'prognosis'

**Web of Science:**

("nutritional status" OR "malnutrition")

AND

("preoperative malnutrition" OR "preoperative dystrophy" OR "preoperative GNRI" OR "preoperative PNI" OR "preoperative CONUT")

AND

"prognosis"

**Cochrane Library:**

("nutritional status" OR "malnutrition")

AND

("preoperative malnutrition" OR "preoperative dystrophy" OR "preoperative GNRI" OR "preoperative PNI" OR "preoperative CONUT")

AND

"prognosis"
