# Supplementary material for: Preoperative nutritional status as a predictor of postoperative overall survival in abdominal tumor surgery: a systematic review and meta-analysis
Source: Front Surg. 2025 Aug 18;12:1645392. doi: 10.3389/fsurg.2025.1645392 (PMC12399654; doi:10.3389/fsurg.2025.1645392)
Supplement: Supplementary file 2 [file Supplementaryfile2.docx]

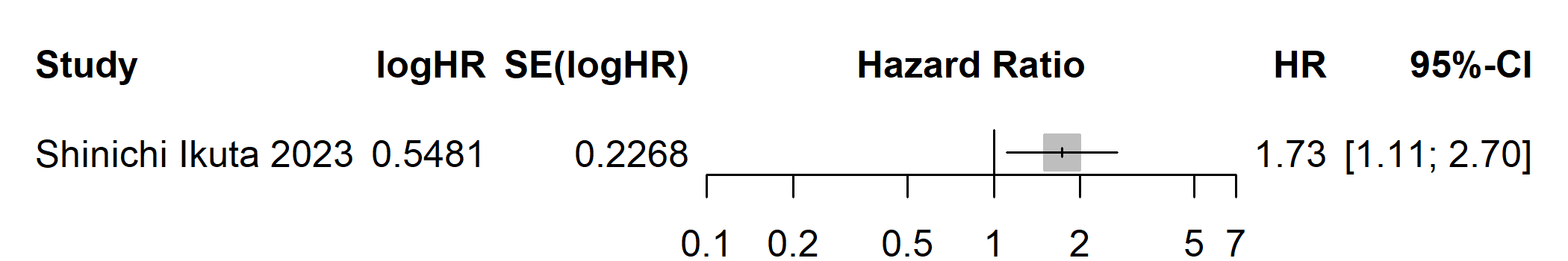


**Figure S1. Forest plot of the association between preoperative nutritional status and OS based on the study focus on cholangiocarcinoma. (OS, overall survival; HR, hazard ratio; Cl, confidence interval).**


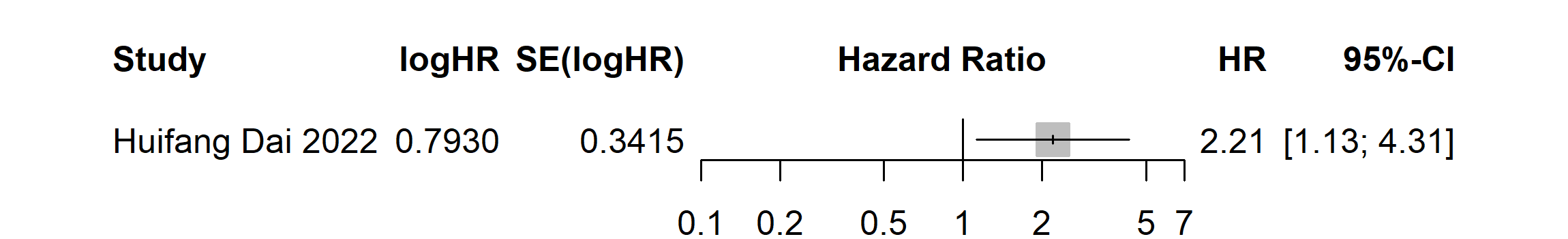


**Figure S2. Forest plot of the association between preoperative nutritional status and OS based on the study focus on gallbladder cancer. (OS, overall survival; HR, hazard ratio; Cl, confidence interval).**


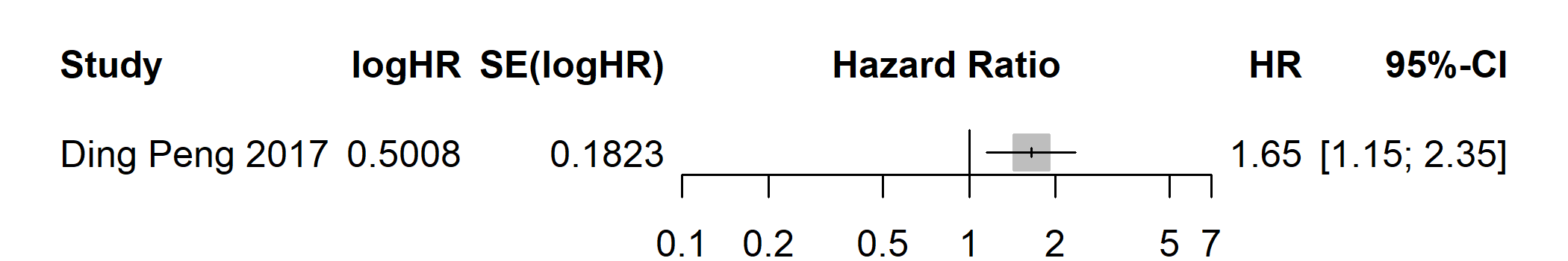


**Figure S3. Forest plot of the association between preoperative nutritional status and OS based on the study focus on renal cancer. (OS, overall survival; HR, hazard ratio; Cl, confidence interval).**
